# Management of an Adenomatoid Odontogenic Tumor Mimicking a Periapical Lesion in a Maxillary Lateral Incisor: A Multidisciplinary Regenerative Approach

**DOI:** 10.7759/cureus.108217

**Published:** 2026-05-04

**Authors:** Jothi Lakshmi Shanmugavelu, Nandhini Palanisamy, Shruthi Venkateshwaran, Vijay Thiyagarajan J

**Affiliations:** 1 Conservative Dentistry and Endodontics, J.K.K. Nattraja Dental College and Hospital, Namakkal, IND; 2 Oral and Maxillofacial Surgery, J.K.K. Nattraja Dental College and Hospital, Namakkal, IND

**Keywords:** adenomatoid odontogenic tumor, enucleation, guided tissue regeneration (gtr), mta-based sealer, orthodontic tooth movement, osseous graft, periapical surgery, platelet-rich fibrin (prf), retreatment, tooth preservation

## Abstract

Periapical radiolucency in endodontically treated teeth is typically managed via conventional retreatment or apical surgery. However, non-inflammatory odontogenic tumors, such as an adenomatoid odontogenic tumor (AOT), can mimic common periapical pathologies, potentially leading to diagnostic errors. This case report describes the multidisciplinary management of an AOT in a 24-year-old male, highlighting the significant diagnostic challenge where a benign tumor mimicked persistent endodontic failure. The patient presented with a 7 × 8 mm hard labial swelling and throbbing pain in the maxillary anterior region. Despite the tooth (12) having undergone previous root canal therapy, clinical and radiographic findings revealed a periapical radiolucency with associated buccal cortical resorption. Unique to this case, the surgical intervention was performed without suspending active orthodontic forces, requiring a regenerative strategy to ensure stability under mechanical loading. The intervention involved non-surgical retrieval of previous filling material, surgical enucleation of the tumor, and advanced regenerative techniques, including osseografts, platelet-rich fibrin, and guided tissue regeneration without root-end resection. This case demonstrates that the synergy of bioactive materials and conservative surgical techniques can achieve predictable bone regeneration and long-term tooth preservation, even in the demanding environment of active orthodontic loading.

## Introduction

The management of periapical radiolucency in endodontically treated teeth typically follows a standard pathway of non-surgical retreatment or apical surgery. However, clinicians must remain vigilant for non-inflammatory pathologies that mimic common endodontic lesions, such as odontogenic tumors. Among these, the adenomatoid odontogenic tumor (AOT) is a benign, slow-growing epithelial lesion that primarily affects the anterior maxilla [1-4]. Although often associated with impacted teeth, a variant known as the follicular type, the rarer extrafollicular variant can present as a well-defined periapical radiolucency associated with erupted, and occasionally previously endodontically treated, teeth.

This diagnostic challenge is significantly amplified when such lesions occur in patients undergoing active orthodontic treatment. The interplay between pathological bone destruction and the mechanical stresses of orthodontic tooth movement creates a complex biological environment. Traditionally, surgical intervention in these cases necessitates a cessation of orthodontic forces to facilitate undisturbed bone healing[5]. However, advancements in regenerative approaches, specifically the use of autologous platelet concentrates and bioactive endodontic materials, have opened new avenues for managing these defects without interrupting orthodontic progress [6,7].

This case report details the successful multidisciplinary management of an extrafollicular AOT in a 24-year-old male. The treatment protocol integrated non-surgical endodontic retreatment with a conservative surgical enucleation that intentionally bypassed apical resection to preserve structural integrity. By utilizing a “sticky bone” regenerative triad comprising an osseous graft, autologous platelet-rich fibrin (PRF), and a guided tissue regeneration (GTR) membrane, effective cortical reconstruction and apical sealing were achieved even under the continuous strain of active orthodontic loading.

## Case presentation

A 24-year-old male presented with a history of severe, throbbing pain and a 7 × 8 mm hard, non-movable labial swelling associated with tooth 12. The patient was in the active phase of fixed orthodontic treatment.

**Figure 1:**
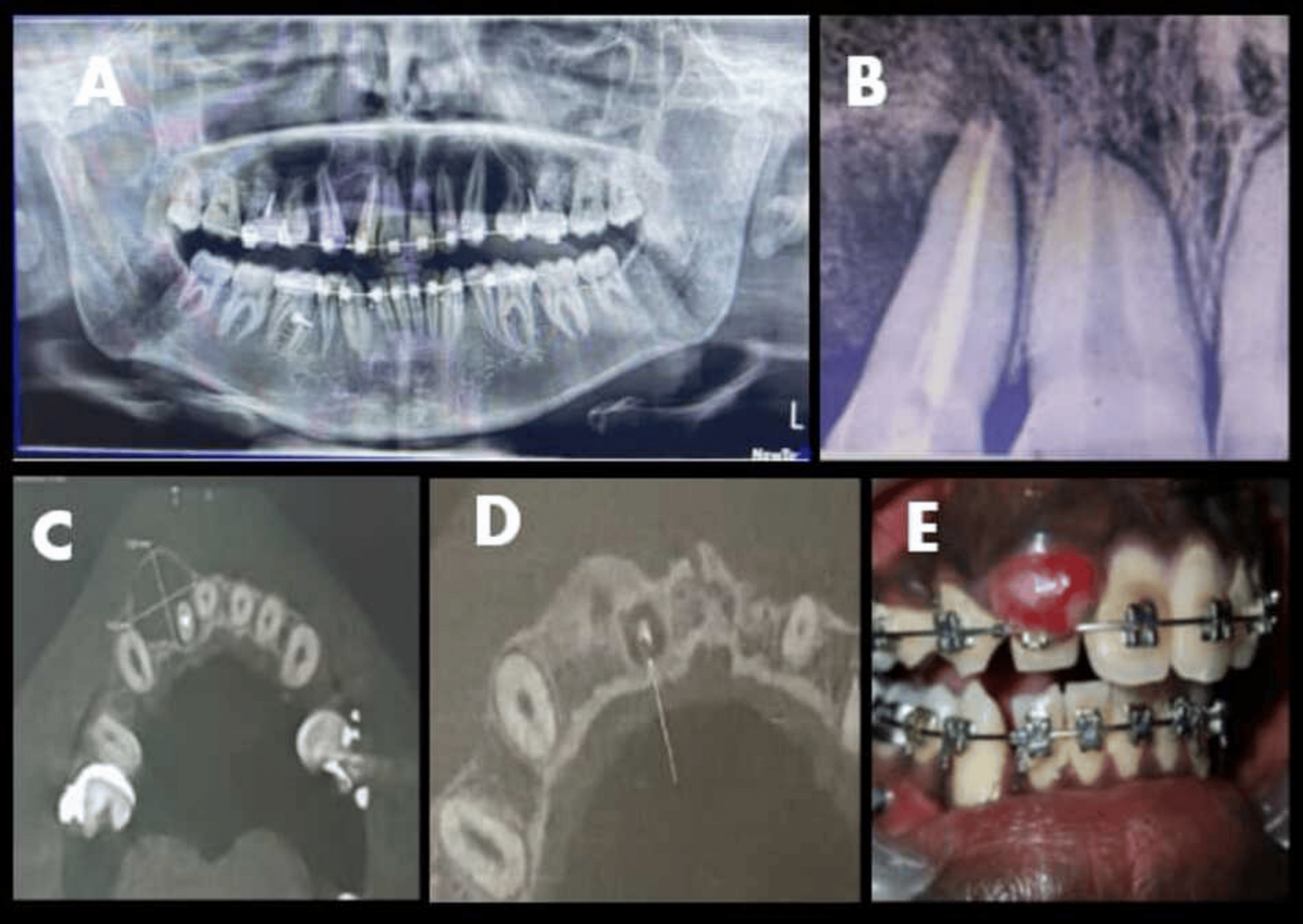
(A) Preoperative orthopantomogram. (B) Periapical radiograph showing a root canal-treated tooth in 12. (C, D) Cone-beam CT images showing expansion of the buccal cortical plate. (E) Intraoral photograph showing swelling in the labial region of 12.

Clinical examination revealed tenderness on percussion and palpation of tooth 12, while adjacent tooth 13 remained vital according to electric pulp testing. Intraoral periapical radiograph revealed a well-defined periapical radiolucency. Despite a history of previous endodontic treatment, the persistence of the lesion and the “hard” nature of the cortical expansion suggested a provisional diagnosis of an extrafollicular AOT. Significantly, due to specific treatment goals and patient requirements, orthodontic forces were not suspended, and the tooth remained tied to the active arch wire during the entire perioperative period (Figure 1).

**Figure 2:**
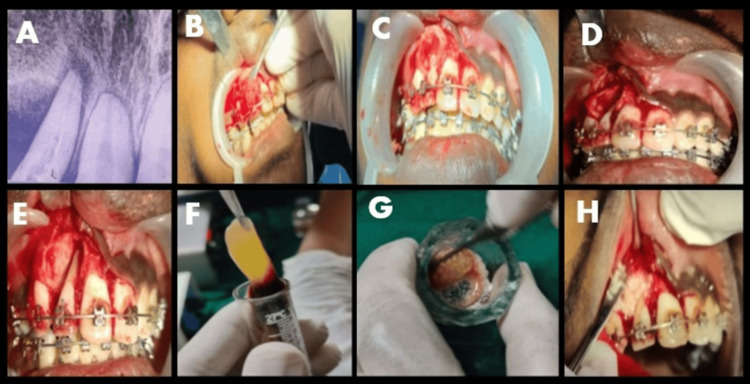
(A) Gutta-percha removed in 12. (B) Flap elevation performed and granulomatous tissues removed. (C) Identification of the buccal cortical defect. (D) Enucleation of the 7 × 8 mm lesion. (E) View of the empty cavity after thorough curettage to healthy, bleeding bone. (F) Platelet-rich fibrin (PRF) prepared. (G) PRF mixed with bone graft and preparation of the sticky bone complex. (H) Bone graft placed in the buccal cortex.

**Figure 3:**
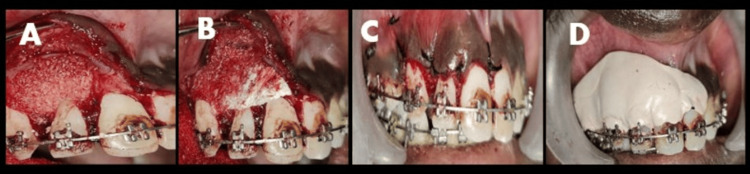
(A) Placement of the bone graft. (B) Final placement of the graft. (C) Suture performed. (D) Placement of Coe-Pak.

**Figure 4:**
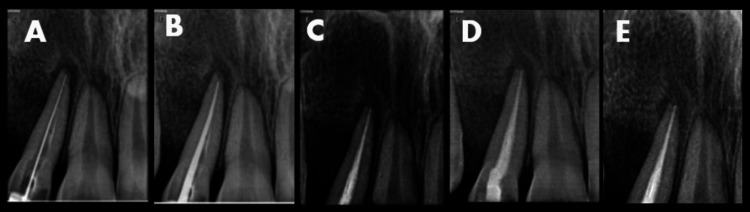
(A) Working length radiograph. (B) Master cone radiograph. (C) Obturation radiograph. (D) One-month review radiograph. (E) Six-month review radiograph.

Under rubber dam isolation, the previous gutta-percha (GP) was retrieved from tooth 12 (Figure 2A). The canal was thoroughly disinfected with 5.25% NaOCl and ultrasonic activation to ensure a sterile environment before surgical entry. Before the surgical intervention, 20 mL of venous blood was drawn from the patient’s antecubital vein and collected in two 10 mL sterile vacuum tubes. These were immediately centrifuged at 2,700 rpm for 12 minutes to obtain autologous PRF. The resulting PRF was utilized to form the “sticky bone” complex and a protective fibrin membrane, ensuring the delivery of concentrated growth factors to the surgical site. Under local anesthesia, a full-thickness mucoperiosteal flap was reflected. Granulomatous tissues were found to be present inside the buccal cortex, which had undergone partial resorption. The lesion, measuring about 7 × 8 mm, was enucleated. Following removal, the internal walls were cauterized to eliminate any residual pathological tissue. Thorough curettage was performed until healthy, bleeding bone was reached (Figures 2B-2E). No apicoectomy (root-end resection) was performed. The root apex was preserved to maintain the maximum crown-to-root ratio, which is critical for the tooth’s stability under future orthodontic loading. To manage the buccal cortical defect, a regenerative approach was employed. Autologous PRF (Figure 2F) was prepared and mixed with an osseograft to create a bioactive “sticky bone” complex optimized for graft stability and enhanced osteoconductive potential. The osseograft-PRF mixture was packed into the defect (Figure 3A). This was followed by the placement of a GTR membrane and a final layer of PRF to act as a biological barrier (Figure 3B). The flap was secured with sutures, and a periodontal dressing (Coe-Pak) was applied to protect the site (Figures 3C, 3D). Following the surgical phase, the tooth was obturated with a cold hydraulic technique using a mineral trioxide aggregate-based sealer to provide a bioactive internal seal promoting hydroxyapatite formation at the apical foramen, demonstrating that even under active orthodontic forces, a combination of bioactive materials and conservative surgical techniques can achieve predictable bone regeneration and long-term tooth preservation (Figure 4).

Following enucleation, the tissue specimen was fixed in 10% formalin and submitted for histopathological examination. Microscopic analysis revealed a well-encapsulated lesion characterized by a proliferation of spindle-shaped or cuboidal epithelial cells forming whorled masses and duct-like structures with a central lumen. Faint areas of eosinophilic amorphous material (dentinoid/pre-enamel matrix) and small foci of calcification were observed throughout the epithelial component. These classic anatomopathological features confirmed the definitive diagnosis of an extrafollicular AOT.

## Discussion

The AOT is a relatively uncommon benign epithelial odontogenic lesion, representing approximately 3% to 7% of all odontogenic tumors [1]. It is classically known as the “two-thirds tumor” because 66% of cases occur in the maxilla, 66% are found in young females, and 66% are associated with an impacted tooth [2]. The extrafollicular variant (Type II) presented here is significantly rarer than the follicular type, accounting for only about 24% of AOT cases [3]. Because it presents as a well-defined radiolucency at the apex of an erupted tooth, it is frequently misdiagnosed as a radicular cyst or a periapical granuloma [4].

Cone-beam CT is the “bridge” between noticing a suspicious X-ray and confirming a surgical plan. It shows the buccal-lingual expansion that two-dimensional X-rays miss. In this case, the previous endodontic treatment of tooth 12 served as a diagnostic mask; however, the “hard” consistency of the labial swelling and the lack of resolution after initial root canal therapy were pivotal clinical indicators that a non-inflammatory pathology was present. A defining complexity of this case is that orthodontic forces were not suspended. Traditionally, periapical surgery necessitates a “healing hiatus” of three to six months [5]. This is because orthodontic tooth movement relies on a delicate balance of pressure-induced bone resorption by osteoclasts and tension-induced bone apposition by osteoblasts. To counteract the risks of active loading and the loss of the buccal cortex, a robust regenerative protocol using PRF was implemented. Performing a regenerative procedure involving a 7 × 8 mm defect while the tooth was under active mechanical strain introduced the risk of graft displacement or fibrous healing instead of osseous regeneration. PRF serves as a second-generation autologous platelet concentrate that creates a fibrin scaffold for growth factor release [6]. Following thorough disinfection, the root canal was obturated using a cold hydraulic technique with MTA Fillapex (Angelus, Londrina, Brazil). This sealer was selected for its proven bioactivity and its capacity to facilitate an apical mineralized barrier, which synergized with the external PRF-GTR regenerative triad to ensure total periapical closure [7]. This is critical because orthodontic tooth movement depends on a delicate balance of bone resorption and tension-induced bone apposition [8]. Recent advancements suggest that controlled mechanical loads may actually enhance bone healing via mechanotransduction [9].

By maintaining the arch wire, the tooth remains stabilized within the arch, which may prevent micro-movements of the root apex within the healing clot, provided the surgical site is adequately protected by a GTR membrane [10]. By mixing the bone graft with PRF, we created “sticky bone” to enhance stability [11], ensuring the graft remains in contact with the internal walls of the defect [12]. As noted in classic pathology texts, AOT is an encapsulated tumor, which facilitates total enucleation [13]. No apicoectomy (root-end resection) was performed. The root apex was preserved to maintain the maximum crown-to-root ratio, which is critical for the tooth’s stability under future orthodontic loading. In AOT, which is an encapsulated tumor and not an infection-driven lesion, the primary goal is total enucleation rather than apical management. Preserving the full root length is biologically advantageous for the orthodontic patient. Resection reduces the root surface area, thereby increasing the concentrated mechanical stress on the remaining periodontium. By keeping the root intact, we ensured a maximum area for the re-establishment of the periodontal ligament, which is essential for the tooth to withstand the tipping and intrusive forces of the active arch wire. To counteract the risks of active loading and the loss of the buccal cortex, a robust regenerative protocol was implemented. PRF serves as a second-generation autologous platelet concentrate. It creates a fibrin scaffold that slowly releases growth factors, including vascular endothelial growth factor and transforming growth factor-beta. These factors are critical for early angiogenesis, which is the rate-limiting step in bone regeneration. By mixing the osseograft with PRF, we created “sticky bone.” This significantly enhances the stability of the graft material, preventing the “washing out” of bone particles and ensuring the graft remains in direct contact with the root and the internal walls of the cauterized defect. The use of a GTR membrane was mandatory to protect the buccal cortical defect. Without a barrier, faster-growing gingival fibroblasts would likely infiltrate the 7 × 8 mm void, leading to soft-tissue invagination rather than true bone formation. The slow release of growth factors from the PRF is the rate-limiting step in this regeneration [14,15], allowing the graft to follow the “PASS” principles for predictable bone formation [16].

The surgical management strictly adhered to the PASS principles for predictable bone regeneration. Primary closure was achieved through tension-free suturing. Angiogenesis was promoted via the growth factor release from the PRF. Space maintenance was ensured by the structural integrity of the osseograft and GTR membrane. Stability of the regenerative complex was maintained through the “sticky bone” technique, which prevented graft displacement despite the active mechanical forces of orthodontic treatment. The tooth was obturated with a cold hydraulic technique using an MTA-based sealer, which complements the external surgical efforts. MTA is highly biocompatible and bioactive; it releases calcium ions that react with phosphate in tissue fluids to form a hydroxyapatite-like layer. This chemical bond at the apical foramen encourages cementogenesis, effectively sealing the tooth from the inside out while the external PRF-bone graft complex seals it from the outside in [17]. This ensures a maximum area for the re-establishment of the periodontal ligament [18], which encourages cementogenesis [19] and provides the necessary grafting support for long-term success [20].

## Conclusions

The management of persistent periapical lesions in the anterior maxilla necessitates a high index of clinical suspicion to effectively differentiate common inflammatory pathologies from rare, non-inflammatory odontogenic tumors, such as the extrafollicular AOT. This case underscores that a conservative surgical approach, prioritizing the preservation of the natural root apex and maintaining the maximum crown-to-root ratio, serves as a viable and often superior alternative to traditional apical resection, especially in patients where structural stability is paramount. The successful outcome of this case was driven by a sophisticated multidisciplinary protocol. By integrating advanced regenerative techniques, specifically the application of a “sticky bone” complex (osseograft mixed with PRF) and GTR, predictable osseous healing and cortical reconstruction were achieved. Remarkably, this regeneration occurred despite the tooth being subjected to the continuous mechanical strain of active orthodontic loading, challenging the traditional requirement for a “healing hiatus.” Ultimately, the synergy between bioactive endodontic materials, such as MTA-based sealers, and robust external regenerative scaffolds provides a stable biological foundation. This approach not only ensures the long-term preservation of the dentition but also allows for the seamless continuation of orthodontic treatment, offering a template for managing complex periapical defects in the modern clinical landscape.
